# Association between Serum Magnesium Levels and Depression in Stroke Patients

**DOI:** 10.14336/AD.2016.0402

**Published:** 2016-12-01

**Authors:** Yingying Gu, Kai Zhao, Xiaoqian Luan, Zhihua Liu, Yan Cai, Qiongzhang Wang, Beilei Zhu, Jincai He

**Affiliations:** Department of Neurology, the First Affiliated Hospital of Wenzhou Medical University, Wenzhou 325000, Zhejinag, China

**Keywords:** magnesium, depression, stroke, biochemical marker

## Abstract

Post-stroke depression (PSD) is a common psychiatric complication of stroke that is associated with a poor outcome in stroke patients. Our aim was to assess the association between the serum magnesium levels and the presence of PSD in Chinese patients. Two hundred nine stroke patients were included in the study. Depressive symptoms were measured by the 17-Hamilton Rating Scale for Depression at 3 months after stroke. Based on the depressive symptoms, diagnoses of depression were made in line with the DSM-IV criteria for PSD. Serum magnesium levels were evaluated using the dimethyl aniline blue colorimetric method at admission. Multivariate analyses were conducted using logistic regression models. Further, 120 normal subjects were recruited, and their serum magnesium levels were also measured as control. At 3 months, fifty-nine patients (28.2%) were diagnosed as PSD. The serum magnesium levels were significantly lower in both PSD patients and non-PSD patients than in normal subjects (*p* < 0.001). Indeed, patients with PSD showed lower serum magnesium levels (*p* < 0.001) than did non-PSD patients at admission. In the multivariate analyses, after adjusting for potential variables, we found that an increased risk of PSD was associated with serum magnesium levels ≤ 0.84mmol/L (OR 2.614, 95% CI 1.178-5.798, *p*=0.018). Low serum magnesium levels at admission were found to be associated with the presence of PSD at 3 months after stroke.

Depression is the most common psychiatric complication encountered by stroke survivors, and affects approximately one third of stroke patients at any time during the follow up [1]. Post-stroke depression (PSD) has a negative impact on outcomes in the forms of greater functional disability, lower quality of life and higher mortality [2, 3].

Magnesium (Mg^2+^) plays a significant role in the physiological function of the brain. Low levels of serum Mg^2+^ have been suggested to play a role in the biological dysregulation contributing to depression. Experimentally induced Mg^2+^ deficiency resulted in depression-like behavior in mice [4], and a recently study suggested that an antidepressant-like activity of Mg^2+^ in an animal model of anhedonia for the first time [5]. In addition, a growing body of evidence indicated an inverse relationship between Mg^2+^ intake and the risk of stroke [6-9].

However, there has been no study on the serum Mg^2+^ levels in patients with PSD. Our aim in this study was to explore the possible link between serum Mg^2+^ levels and the occurrence of depression at 3 months after stroke.

## MATERIALS AND METHODS

Two hundred nine patients admitted to the stroke unit, the First Affiliated Hospital, Wenzhou Medical University, were prospectively included in the study during the period from October 2013 to September 2014. Patients from 18 to 80 years of age with an acute ischemia stroke were included. The exclusion criteria were: severe cognitive impairment, severe aphasia, patients with a history of psychiatric disorders, renal insufficiency, metabolic abnormalities, significant acute medical illness and significant acute neurological illness. Patients taking Mg^2+^ supplementation were also excluded. The control cases (N = 120) were recruited from a health survey in a community of similar age and gender distribution to the acute ischemia stroke patients. The control cases had no a history of psychiatric illness or neurological disorders, and their score on the 17-Hamilton Scale was less than seven. The study protocol was approved by the Medical Ethics Committee of the First Affiliated Hospital of Wenzhou Medical University. Written informed consent was obtained from the participants or their nearest relatives.

**Table 1:** Baseline clinical characteristics in patients with and without post-stroke depression at 3-months.

	Non-PSD (n = 150)	PSD (n = 59)	Control (n = 120)
Demographic characteristics			
Age (years), mean ± SD	60.41±10.63	62.39 ± 9.66	59.47 ± 9.35
Female (%)	39(26.0)	30 (50.8)^b^ ^d^	39 (32.5)
BMI (kgm^-2^), mean ± SD	24.00±3.13	24.69 ± 3.32	
Education (years), median (IQR)	5(1-7)	3 (0-7)	
Vascular risk factors (%)			
Hypertension	139(92.7)	54 (91.5)	
Diabetes mellitus	41(27.3)	29 (49.2)^b^	
Hyperlipidaemia	72(48.0)	33 (55.9)	
Coronary heart disease	4(2.7)	4 (6.8)	
History of stroke	15(10.0)	8 (13.6)	
Current smoking	57(38.3)	15 (27.8)	
Alcohol consumption	61(41.5)	15 (27.8)	
Systolic blood pressure (mmHg), mean ± SD	157.12±22.78	160.63 ± 22.52	
Diastolic blood pressure(mmHg), mean ± SD	86.90±13.37	84.31 ± 12.17	
Lesion location			
Frontal lobe	33 (22.0)	11 (18.6)	
Parietal lobe	29 (19.3)	13 (22.0)	
Temporal lobe	17 (11.3)	8 (13.6)	
Occipital lobe	20 (13.3)	7 (11.9)	
Basal ganglia	62 (41.3)	27 (45.8)	
Brainstem	23 (15.3)	11 (18.6)	
Cerebellum	7 (4.7)	4 (6.8)	
Other	78 (52.0)	30 (50.8)	
Neuropsychological function			
NIHSS at admission, median (IQR)	3 (1-4)	3.5 (2-6)^b^	
mRS at discharge, median (IQR)	1 (1-2)	2 (1-3)^b^	
Mg^2+^ (mmolL^-1^), median (IQR)	0.88 (0.84 - 0.94)^d^	0.84 (0.77-0.87)^a^^c^	0.96 (0.84 - 1.07)
Mg^2+^ (mmolL^-1^), mean ± SD	0.89 ± 0.10^d^	0.83 ± 0.09^a^^c^	0.94 ± 0.13

Results were expressed as percentages or as medians (IQR) and means (SD). SD: standard deviation; IQR: interquartile range; BMI: body mass index; NIHSS: National Institutes of Health Stroke Scale; mRS: modified Rankin Scale; Mg^2+^: Magnesium; PSD: post-stroke depression;

a *p* < 0.001 compared with non-PSD;

b *p* < 0.05 compared with non-PSD;

c *p* < 0.001 compared with controls;

d *p* < 0.05 compared with controls.

Depression assessments were performed by a neurologist/psychiatrist who was blind to the laboratory result of the stroke patients at 3 months after stroke [10, 11]. Stroke survivors finished the 17-Hamilton Rating Scale for Depression (HAMD) at the 3-month follow-up [12]. Patients with a Hamilton Depression Scale score > 7 were given the Diagnostic and Statistical Manual of Mental Disorders, fourth edition (DSM-IV) criteria for diagnosis of PSD. The serum Mg^2+^ levels were evaluated using the Dimethyl aniline blue colorimetric method with a Beckman Coulter AU5800 automatic analyzer. The Mg^2+^ levels were categorized into three groups according to tertiles: ≤ 0.84 mmol/L, 0.85-0.90 mmol/L and ≥ 0.91 mmol/L. The influence of the Mg^2+^ levels on PSD was estimated by binary logistic regression analysis, after adjusting for potential confounding variables. Statistical significance was defined as *p* < 0.05.

## RESULTS

In the study population, 33.0% were females, and the average age was 60.97 ± 10.39 years. The median (quartiles) NIHSS score at admission was 3 (1-4). The baseline characteristics of the two groups are described in Table 1. Fifty-nine patients (28.2%) showed depression at 3 months after admission. The results indicated that the median serum Mg^2+^ levels were significantly different among PSD patients, non-PSD patients and normal subjects (*p*<0.001). Indeed, the median serum Mg^2+^ levels were significantly lower in PSD patients than in non-PSD patients (*p*<0.001). Significant differences were observed between the PSD and non-PSD groups in the Mg^2+^ level tertiles of patients (*p* = 0.013) (Table 2).

**Table 2:** Magnesium levels tertiles of patients

	Non-PSD (n=150)	PSD (n=59)	*p*
Mg^2+^ category, n (% of total population)			0.013
Low tertile (≤0.84mmol/L)	45(30.0%)	30(50.8%)	0.005
Intermediate tertile (0.85-0.89 mmol/L)	59(39.3%)	19(32.2)	0.337
High tertile (≥0.90mmol/L)	46(30.7%)	10(16.9%)	0.044

In the multivariate Logistic regression analysis, with the intermediate tertile taken as reference, the low tertile of Mg^2+^ level (≤0.84 nmol/l) was independently associated with the development of PSD (OR 2.614, 95% CI 1.178-5.798, *p*=0.018) after adjusting for the above potential confounders, whereas the high tertile of Mg^2+^ levels (≥0.91 mmol/l) with an OR 0.579 (95%CI 0.225-1.494, *p*=0.259). Moreover, gender and mRs at discharge were significantly associated with the development of PSD in acute ischemia stroke patients (Table 3).

**Table 3:** Multivariate logistic model of the clinical determinants of PSD

Variables	OR (95% CI)	*p*
Magnesium		0.006
Low tertile	2.614 (1.178-5.798)	0.018
High tertile	0.579 (0.225-1.494)	0.259
sex	3.447 (1.702-6.981)	0.001
NIHSS on admission	1.151 (0.969-1.369)	0.110
mRS at discharge	1.534 (1.033-2.278)	0.34
DM	1.495 (0.734-3.044)	0.267

PSD: post-stroke depression; OR: odds ratio; CI: confidence interval; NIHSS: National Institutes of Health Stroke Scale; mRS: modified Rankin Scale; DM: Diabetes mellitus

## DISCUSSION

To the best of our knowledge, this is the first study to investigate the relationship between the serum Mg^2+^ levels and the post-stroke depression. Our study demonstrated that low serum Mg^2+^ was associated with the development of depression at 3 months after stroke, indicating the possible involvement of the NMDA receptors in this activity. To our knowledge, neuronal Mg^2+^ levels are highly important in the regulation of NMDA receptor excitability. In Mg^2+^ deficiency, the NMDA receptors become hyperexcitable [13], which means that fewer NMDA channels will be blocked. In contract, when the Mg^2+^ levels in the central nervous system (CNS) are low, the γ-aminobutyric acid (GABA) receptors are less stimulated [14], whose function is also regulated by Mg^2+^, which in turn relieves the Mg^2+^ block of the NMDA receptor. The final mechanism contributing to the hyperexcitability of NMDA-receptor abundant neurons is the inhibition of glutamate release from the presynaptic neuron, which leads to depressive behavior. One study found that NMDA receptor blockade at rest triggers rapid behavioral antidepressant response [15]. Several studies have proposed that Mg^2+^ may relieve depression by blocking the NMDA receptor, whose dysfunction is a major causative factor in depression pathology [16]. As a result of the described mechanisms, the Mg^2+^ deficiency caused by NMDA receptor hyperexcitability may contribute to the occurrence of PSD.

Several limitations of this study should be noted. First, the serum Mg^2+^ levels were assessed only once at admission. Second, the study was performed in only one clinical institute. In addition, patients who had more serious stroke died before the 3-month follow up were not included. Some patients who died and had depression might have been excluded.

### Conclusion

The present study showed that, low serum Mg^2+^ levels at admission are associated with the development of depression at 3 months after stroke. Serum Mg^2+^ level alterations may play an important role in the presence of depression 3 months after stroke.
